# Whole grain consumption trends and associations with body weight measures in the United States: results from the cross sectional National Health and Nutrition Examination Survey 2001–2012

**DOI:** 10.1186/s12937-016-0126-4

**Published:** 2016-01-22

**Authors:** Ann M. Albertson, Marla Reicks, Nandan Joshi, Carolyn K. Gugger

**Affiliations:** 1 James Ford Bell Technical Center, General Mills Bell Institute of Health and Nutrition, 9000 Plymouth Avenue North, Minneapolis, MN 55427 USA; 2 Department of Food Science and Nutrition, University of Minnesota, 1334 Eckles Avenue, St. Paul, MN 55108 USA; 3 General Mills India Pvt. Ltd, 601-Prudential, Hiranandani Business Park, Powai, Mumbai 400076 India

**Keywords:** Total grain consumption, Whole grain consumption, Food sources, Adiposity measures, Body mass index, Cross-sectional survey

## Abstract

**Background:**

The purpose of this study was (1) to describe intakes of total grain and whole grain in the United States over the past 12 years and major dietary sources, and (2) to determine the relationship between whole grain intake and adiposity measures for children and adults.

**Methods:**

Cross-sectional dietary data from the continuous National Health and Nutrition Examination Survey 2001–12 (6 2-year cycles) for children 6–18 years (*n* = 15,280) and adults 19+ years (*n* = 29,683) were linked to the My Pyramid and Food Patterns Equivalents Databases to assess daily intake of total grain and whole grain. These populations were classified into groups based on average whole grain intake: 0 ounce equivalents (oz eq)/day, > 0 and <1 oz eq/day, and ≥1 oz eq/day. Within these classifications, body mass index, waist circumference, and percent overweight/obese were identified. Regression and logistic regression analyses were used to assess associations between these dependent variables and whole grain intake.

**Results:**

Adults consumed a mean 0.72 whole grain oz eq/day in 2001–02 and 0.97 oz eq/day in 2011–12 and children consumed a mean 0.56 whole grain oz eq/day in 2001–02 and 0.74 oz eq/day in 2011–12. While over 70 % of children and 60 % of adults met daily intake recommendations for total grain, less than 1.0 and 8.0 % percent of children and adults, respectively, met whole grain recommendations in 2011–12. Adults and children who consumed whole grain had significantly better intakes of nutrients and dietary fiber compared to non-consumers. From 2001 to 2012, grain mixed dishes and yeast breads were the leading sources of total grain, while yeast breads and ready to eat cereals were the leading sources of whole grain for both children and adults. Multiple regression analysis showed a significant, inverse relationship between body mass index and waist circumference with respect to whole grain intake after adjustment for covariates in both children and adults (*p* < 0.05). Similarly, logistic regression analysis showed a significant inverse relationship between percent overweight/obese and whole grain intake (*p* < 0.05).

**Conclusions:**

Although most children and adults meet daily intake goals for grain foods overall, whole grain as a portion of total grain intake continues to be consumed at levels well below recommendations. The data from the current study suggest that greater whole grain consumption is associated with better intakes of nutrients and healthier body weight in children and adults. Continued efforts to promote increased intake of whole grain foods are warranted.

## Background

Current U.S. dietary guidance includes a recommendation that at least half of all grains be consumed as whole grains (WG) [1] with the total amount of grains dependent on the individual’s age, sex, and level of physical activity. This recommendation is based on reviews of prospective studies and randomized trials indicating that WG intake among adults is associated with chronic disease risk reduction including type 2 diabetes, coronary heart disease, hypertension, and lower risk of weight gain [2–9] and lower all-cause mortality [10]. The protective effects of WG intake have been attributed mostly to bran and germ components, including bioactive compounds such as antioxidants and minerals [2, 11, 12].

National Health and Nutrition Examination Survey (NHANES) data from 1999 to 2004 showed an inverse relationship between WG intake and measures of adult adiposity including body mass index (BMI), waist circumference and percent overweight/obese (OW/OB) [13, 14]. However, an inverse relationship between WG intake and measures of adiposity among children and adolescents has been reported less consistently. For example, after adjusting for food group intake, WG intake was not associated with BMI, waist, arm or thigh circumference among adolescents measured as part of the NHANES data collection from 1999 to 2004 [15]. However, an inverse association was reported between intake at the level of 1.5 to < 3 daily WG servings and BMI z-score and BMI-for-age percentile among adolescents based on NHANES data collected during the same time period [16].

Greater intake of WG foods has also been associated with improved diet quality among children, adolescents and adults in the U.S. based on NHANES data (1999–2004) and in the United Kingdom based on national data [15, 17–19]. Greater WG intake has been associated with higher intakes of fiber, folate, magnesium, phosphorus, iron, vitamins A, E and select B vitamins, as well as energy and lower intakes of saturated fat and cholesterol, added sugars and sodium. Several of these nutrients (vitamin A, vitamin E, folate, magnesium) have been identified as underconsumed by the general public in the U.S. [20].

NHANES data (2009–10) indicate that only 2.9 and 7.7 % of children/adolescents and adults consumed ≥ 3 WG ounce equivalents (oz eq)/day, respectively [21], consistent with estimates from earlier NHANES data (1999–2004) [17, 18]. Efforts to increase WG intake by Americans include an emphasis on consumption of WG foods in dietary guidance since 2005 [1, 20]. The focus on WG foods fueled consumer interest and demand, as national survey results in 2015 indicate two-thirds of consumers considered the WG content of a food an important factor when making purchase decisions [22]. Following consumer demand, the availability of WG foods has expanded in the marketplace and food manufacturers have conducted applied research to address barriers such as limited availability and perceived poor palatability of WG products [23]. In 2011, 3378 new WG food products were introduced compared to 855 in 2005 (a 4-fold increase) [24]. The alignment of several nutrition assistance programs with dietary guidance regarding WG intake has also resulted in increased product availability in the marketplace. For example, the Women, Infants and Children Special Supplemental (WIC) Program revised the food package provided to participants in 2009 to authorize inclusion of whole wheat/whole grain bread and other WG options [25]. Several regional studies have shown that the revised WIC food package resulted in improved availability of WG foods in convenience and grocery stores [26, 27].

WG food sources were reported for children/adolescents and adults in the U.S. using national dietary intake data from 2009 to 10 [21]. Primary sources included ready to eat (RTE) cereals, yeast bread and rolls, oatmeal and popcorn with adults consuming slightly more oatmeal than children/adolescents (21 % of the total WG intake vs. 12 %) and less RTE cereals than children/adolescents (20 % of the total WG intake vs. 25 %). Recent trends in breakfast choices among consumers, and the addition of new products to the marketplace may have resulted in changes in WG food sources over time. Identification of the main sources of total grain (TG) consumed by adults and children may help determine additional foods that could be reformulated to include more whole grains.

A comprehensive report based on data from USDA’s 1994–96 Continuing Survey of Food Intakes by Individuals was published in 2000 providing the first population estimates of TG and WG intake, major dietary sources in the U.S. and impact on diet quality [28]. Over the past 20 years, new research on health benefits as well as changes in dietary guidance, consumer interest, and availability of WG foods indicates that an examination of changes in TG and WG intake and diet quality, and dietary sources is warranted. Recently McGill et al. [29] reported 10-year trends in WG intake and a descriptive analysis of WG food sources for U.S. adults and children based on NHANES data 2001–2010, however, this report does not include the latest dietary intake information available from NHANES 2011–2012. A re-examination of the role that WG intake may play in adiposity is also warranted given the inconsistent reports of relationships between WG intake and weight status among children/adolescents [15, 16]. Therefore, the purpose of the current study was 1) to describe how daily intakes and primary dietary sources of TG and WG have changed over the past 12 years, and 2) to test the hypotheses that an inverse association exists between WG intake and measures of adiposity among children (6–18 y) and adults (19+ y). This article reports on trends in TG and WG intake over time in the US population, whether the trends reflect dietary recommendations, and health benefits related specifically to WG intake on improvements in nutrient intake and body weight.

## Methods

### Study population

Data from the NHANES 2001–2012 were used for the present analysis [30]. The continuous NHANES is a cross-sectional survey that collects data about the nutrition and health status of the U.S. population using a complex, multi-stage, probability sampling design [30]. NHANES is conducted in a non-institutionalized, civilian U.S. population by the National Center for Health Statistics (NCHS). NHANES participants completed a comprehensive questionnaire assessing dietary behaviors, health history, socioeconomic status, and demographic information at NHANES Mobile Examination Centers and in participant’s homes. The NCHS Research Ethics Review Board reviewed and approved all study protocols for NHANES 2001–12.

### Dietary intake assessment

Trained interviewers conducted in-person 24-h dietary recalls using the USDA’s Automated Multiple-Pass Method (AMPM) 5-step data collection [31]. Dietary data included detailed descriptions of all food and quantities eaten. Two days of dietary intake were collected from participants. Dietary intake data for the first day were collected through in-person interview. On the second day, dietary intake data were collected by telephone interview. Detailed descriptions of the dietary interview methods are provided in the NHANES Dietary Interviewer’s Training Manual, which includes pictures of the Computer-Assisted Dietary Interview system screens, measurement guides, and charts used to collect dietary information [32].

The current study utilized intake data only from the first day of data collection unless otherwise noted. Participants with complete and reliable dietary data were included, as determined by the NCHS. USDA’s Food and Nutrient Database for Dietary Studies (FNDDS) was used to code and estimate the nutrient content of reported food and beverages for each respective 2 year cycle of NHANES from 2001 to 12 [33].

### Grain intake and main food sources

Mean daily intake of TG and WG reported in ounce equivalents (oz eq) and the food sources consumed were calculated for children 6–18 years old and adults 19 years and above for each 2 year cycle of NHANES from 2001 to 02 to 2011–12. USDA’s My Pyramid equivalent database (MPED) for USDA survey foods, 1994–2002, Version 1.0 and 2003–2004, version 2.0 A (MPED 1 and 2) were used to calculate TG (refined grain + WG) and WG intake for NHANES 2001–2004 participants [34, 35]. USDA’s Food Pattern Equivalent Database, (FPED) 2005–2006, FPED 2007–2008, FPED 2009–2010 and FPED 2011–2012 were used to calculate TG (refined grain + WG) and WG intake for NHANES 2005–2012 participants [36–39]. MPE food data files from 2001 to 2004 contain the number of servings per 100 g of food for 32 MyPyramid food groups, three of which are WG, refined grain, and TG. The FPED database was revised in 2005 to report grain consumption in terms of ounce equivalents (oz eq) in order to align with the food group serving definitions used in the 2005 Dietary Guidelines for Americans. Examples of grain food servings contained within the database include one slice of bread, a cup of cereal, or ½ cup of hot cereal, cooked pasta, rice, or other grain such as bulgur, oatmeal, and cornmeal. Grain components contributing to total WG intake were defined as including all parts of the entire kernel (germ, bran and endosperm) such as oatmeal, popcorn, whole wheat, whole barley, wild rice and quinoa [36–39].

National Cancer Institute’s (NCI) Usual Intake method was used to estimate percent of individuals meeting the recommendations of at least 6 oz eq of TG and at least 3 oz eq of WG per day [40]. Two 24-h dietary recalls (day 1 and 2) were used to estimate these numbers. This analysis was done for each 2 year cycles of NHANES from 2003 to 2012. For NHANES 2001–02, only one 24-h dietary recall data was available, therefore it was not possible to estimate the percent meeting the recommendation of TG or WG for the year 2001–02.

### Nutrient intake by whole grain consumption

The NHANES 2001–12 were used in this secondary analysis to examine the relationship between WG intake and nutrient intake among children (6–18 y of age) (*n* = 15,280) and adults (≥19 y of age) (*n* = 29,683). Participants were categorized into one of three WG consumption groups based on oz eq consumed per day: none or no WG (0 oz eq/day), low (>0 to <1 oz eq/day), and high (≥1 oz eq/day). Intake of energy, macronutrients and selected micronutrients were calculated, and do not include contributions from dietary supplements, medications, or plain drinking water. The mean daily nutrient intakes are compared to understand if any significant difference exists between the three WG groups.

### Whole grain intake and adiposity measures

Adiposity status was assessed by using anthropometric measurements (i.e., weight, height, and waist circumference) conducted by NHANES personnel in the Mobile Examination Center [41]. BMI was calculated as kg/m^2^. Status of overweight and/or obesity (%) was assigned by two different methods for children and adults. Among adults (≥19 y of age), individuals with BMI ≥ 25 are defined as overweight/obese (OW/OB) and those with BMI ≥ 30 are defined as obese [42]. Among children (6–18 y of age), individuals with BMI at or above the 85th percentile for children of the same age and sex are defined as OW/OB and those with BMI at or above the 95th percentile are defined as obese [43]. For NHANES 2001–12, the relationship between WG intake and adiposity measures were studied as a continuous sample.

### Statistical analysis

All statistical analyses were performed with SAS 9.2 (SAS Institute, Cary, NC). Dietary intake sample weights were applied to all analyses to account for the unequal probability of selection, non-coverage, and non-response bias resulting from oversampling of low-income persons, adolescents, elderly persons, African-Americans, and Mexican-Americans. Demographic, socioeconomic, and physical activity information was obtained from their respective NHANES questionnaires. Mean TG or WG (oz eq/d) intakes were calculated using PROC SURVEYMEANS for all the 2 year cycles of NHANES from 2001 to 12 to understand the trend among children and adults. Regression analysis was performed to understand the linear and curvilinear trend in mean TG or WG intake across different years of NHANES. Analysis of Variance (ANOVA) was performed to compare mean TG or WG intake of every cycle of NHANES with 2011–12. Mean TG or WG intake from different food sources were estimated using PROC SURVEYMEANS and percent contribution from different food groups were calculated as a result. This analysis was performed separately for each cycle of NHANES from 2001 to 12 to understand the change in the sources of grain intake over the period of 12 years. The NCI usual intake method was used to estimate the percent individuals meeting the dietary recommendation of at least 6 servings of TG or at least 3 servings of WG daily for each cycle of NHANES from 2003 onwards. ANOVA was used to compare the mean nutrient intake between three WG consumption groups: none or no WG (0 oz eq/day), low (>0 to <1 oz eq/day), and high (≥1 oz eq/day). Baseline age and weight characteristics were estimated for each NHANES cycle using the appropriate survey weights. Multivariate linear regression was performed to determine the extent to which adiposity measures (BMI, and waist circumference) were explained by WG intake. Similarly, multivariate logistic regression was performed to estimate how the likelihood of being overweight/obese or obese, respectively, differs across WG consumption groups among children (6–18 y of age) and adults (≥19 y of age). The analysis was adjusted for the following covariates: age, age^2^, gender, race/ethnicity, total calorie intake (kcal), alcohol intake and physical activity. A *P* value of ≤0.05 was considered statistically significant.

## Results

Mean daily TG intake in the U.S. population has remained relatively stable from 2001 to 2012 for both adults and children, whereas mean daily WG intakes in the U.S. population have increased slightly (Table 1). According to NHANES 1-day records, children consumed an average of 7.2 oz eq/day of TG in 2011–12, a significant decrease from 7.4 oz eq/day reported in 2001–02. In adults, mean TG intake decreased from 6.9 oz eq/day in 2001–02 to 6.4 oz eq/day 2007–08, but then increased to 6.8 oz eq/d in 2011–12 (*p* = 0.0041 for curvilinear trend).

**Table 1:** Daily intake of total grain and whole grain ounce equivalents based on NHANES data (2001–02 to 2011–2012)^a,b,c,d^

Age-group	2001–2002	2003–2004	2005–2006	2007–2008	2009–2010	2011–12	*P* for linear trend	*P* for curvilinear trend
Total grain (oz eq/d)								
	Mean ounce equivalent ± standard error^b^							
*n*	3147	2747	2838	2134	2263	2151		
6–18 years	7.4 ± 0.1	7.5 ± 0.1	7.4 ± 0.1	6.8 ± 0.2	7.2 ± 0. 2	7.2 ± 0.2	0.0476	0.3292
*n*	4647	4456	4405	5477	5820	4878		
19+ years	6.9 ± 0.1	6.8 ± 0.1	6.7 ± 0.1	6.4 ± 0.1*	6.5 ± 0.1*	6.8 ± 0.1	0.1513	0.0041
Whole grain (oz eq/d)								
	Mean ounce equivalent ± standard error^b^							
*n*	3147	2747	2838	2134	2263	2151		
6–18 years	0.56 ± 0.03*	0.49 ± 0.03*	0.49 ± 0.04*	0.53 ± 0.04*	0.61 ± 0.02*	0.74 ± 0.03	<0.0001	<0.0001
*n*	4647	4456	4405	5477	5820	4878		
19+ years	0.72 ± 0.03**	0.63 ± 0.03**	0.73 ± 0.03**	0.69 ± 0.04**	0.84 ± 0.04**	0.97 ± 0.05	<0.0001	0.0007

^a^Day 1 from foods only, excluding pregnant and breastfeeding women

^b^Mean Total Grain (oz eq/d) and Whole Grain (oz eq/d) intake were calculated using PROC SURVEYMEANS for all the 2 year cycles.

^c^Regression analysis was performed to understand the linear and curvilinear trend in mean Total Grain and Whole Grain intake across different years of NHANES

^d^ANOVA was performed to compare mean Total Grain and Whole Grain intake of every cycle of NHANES with 2011–12

*Mean Total Grain intake is significantly different than mean Total Grain intake of 2011–12 (p < 0.05)

**Mean Whole Grain intake is significantly different than mean Whole Grain intake of 2011–12 (*p* < 0.05)

In comparison, mean WG intake in adults was 0.72 oz eq/day in 2001–02 and an average of 0.97 oz eq/d in 2011–12, a 35 % increase in WG intake over the 12-year time period (Table 1). The mean WG intake in 2011–12 was significantly greater than all previous waves. Children age 6–18 consistently consumed slightly lower levels of WG than adults over the 12-year time period: on average, 0.56 oz eq/d in 2001–02 and 0.74 oz eq/d in 2011–12, a 32 % increase over 12 years. Similar to adults, mean intake in 2011–12 was significantly greater than all previous waves. Based on 2011–12 consumption levels, WG represented 10 % of mean TG intake in children, and 14 % of mean TG intake in adults, which is an increase from 7.5 % in children and 10 % in adults in 2001–02. Forty six percent of adults and children reported consuming zero oz eq/d of WG in the continuous sample from NHANES 2001–12 (Tables 5 and 6).

Over 74 % of children and 60 % of adults reported meeting the daily recommendation for TG intake (at least 6 oz eq/d) in 2011–12, which was similar to values reported in 2003–04 (Table 2). In comparison, the percent of adults meeting recommendations for WG (3 oz eq/day) has increased steadily since 2003–04. Despite these increases, only 7.9 % of adults consumed the recommended amount of WG in 2011–12 based on 2-day diet records. Less than 0.5 % of children age 6–18 met the WG recommendations in any of the years evaluated.

**Table 2:** Percent individuals meeting their daily recommendation of total grain and whole grain ounce equivalents based on NHANES (2003–04 to 2011–12)^a,b^

Age-group	2001–02	2003–04	2005–06	2007–08	2009–10	2011–12
Total grain (6 oz eq/d)						
	Percent meeting (%)^b^					
*n*	3147	2747	2838	2134	2263	2151
6–18 years	NA	73.7	70.3	61.7	71.9	74.8
*n*	4647	4456	4405	5476	5820	4878
19 + years	NA	60.6	56.9	54.1	56.1	60.9
Whole grain (3 oz eq/d)						
	Percent meeting (%)^b^					
*n*	3147	2747	2838	2134	2263	2151
6–18 years	NA	0.18	0.17	0.03	0.04	0.25
*n*	4647	4456	4405	5476	5820	4878
19 + years	NA	1.9	2.6	1.6	4.5	7.9

^a^Day 1 and 2 from foods only, excluding pregnant and breastfeeding women

^b^The NCI usual intake method was used to estimate the percent of individuals meeting the recommendation of at least 6 ounce equivalents of total grain and 3 ounce equivalents of whole grain per day for each cycle of NHANES from 2003 onwards

Table 3 shows the % TG intake from various food groups from 2001 to 2012 for each age group. Yeast breads, grain mixtures (including frozen meals and soups), crackers/salty snacks and other grain foods were consistently the leading sources of TG intake in children and adults. For children, the largest increase in TG intake over the 12-year period was from grain mixtures (23.3 to 30.9 %), with decreases in the percent contribution from yeast breads, pasta/rice and RTE cereals (Table 3). A similar trend was seen in adults, where relative TG intake from grain mixtures increased 7.1 % across 2001–12, with slight decreases in the contribution from yeast breads, quick breads and pasta/rice (Table 3).

**Table 3:** Percent total grain intake from different food groups in children (6–18 years) and adults (19+ years) based on NHANES (2001–02 to 2011–12)^a,b^

Children (6–18 years)	2001–02 (*n* = 3147)	2003–04 (*n* = 2747)	2005–06 (*n* = 2838)	2007–08 (*n* = 2134)	2009–10 (*n* = 2263)	2011–12 (*n* = 2151)
Food	Percent (%)^b^					
Grain mixtures, frozen plate meals, soups, meat substitutes	23.3	26.1	29.9	25.4	26.5	30.9
Yeast breads, rolls	23.0	24.8	22.2	21.6	22.5	20.8
Crackers and non-popcorn salty snacks from grains	11.7	10.8	10.7	12.8	11.6	10.8
All other foods^c^	10.9	12.5	10.4	11.3	10.2	10.6
Cakes, cookies, pies, pastries	8.7	8.9	8.8	8.5	7.8	8.9
RTE cereals	6.9	6.0	4.8	4.9	4.5	5.0
Pastas, macaroni, rice	5.8	2.2	2.3	3.0	3.7	3.3
Quick breads	4.7	4.2	6.2	7.3	7.9	4.8
Pancakes, waffles, french toast, crepes	2.9	2.8	3.3	3.4	3.8	3.2
Popcorn	1.0	1.2	0.9	0.8	1.0	1.1
Oatmeal	0.7	0.2	0.4	0.7	0.4	0.5
Other cooked cereals	0.4	0.2	0.2	0.1	0.1	0.2
Flour and dry mixes	0.0	0.0	0.0	0.0	0.0	0.0
Total Grain	100.0	100.0	100.0	100.0	100.0	100.0
Adults (19+ years)	2001–02 (*n* = 4647)	2003–04 (*n* = 4456)	2005–06 (*n* = 4405)	2007–08 (*n* = 5476)	2009–10 (*n* = 5820)	2011–12 (*n* = 4878)
Food	Percent (%)^b^					
Yeast breads, rolls	29.2	27.6	27.0	27.9	29.3	26.3
Grain mixtures, frozen plate meals, soups, meat substitutes	17.4	21.7	23.6	20.9	20.9	25.4
All other foods^c^	12.4	13.0	10.8	11.5	11.1	11.8
Crackers and non-popcorn salty snacks from grains	8.8	8.1	8.7	9.6	8.3	8.4
Cakes, cookies, pies, pastries	8.3	8.9	8.3	7.9	7.4	8.6
Quick breads	8.2	7.6	9.3	9.7	9.6	6.3
Pastas, macaroni, rice	5.8	4.0	3.7	4.3	4.4	4.5
RTE cereals	4.8	4.0	4.2	4.3	4.4	4.3
Pancakes, waffles, french toast, crepes	1.8	1.9	2.0	1.6	1.6	1.5
Oatmeal	1.4	1.5	1.3	1.3	1.4	1.5
Popcorn	1.2	1.0	0.9	0.8	1.0	0.8
Other cooked cereals	0.7	0.5	0.4	0.3	0.5	0.5
Flour and dry mixes	0.0	0.0	0.0	0.0	0.0	0.0
Total Grain	100.0	100.0	100.0	100.0	100.0	100.0

^a^Day 1 from foods only, excluding pregnant and breastfeeding women

^b^Mean Total Grain intake from different food sources were estimated using PROC SURVEYMEANS and percent Total Grain contribution from different food groups were calculated

^c^Meat, poultry, fish mixtures including sandwiches

Yeast breads and RTE breakfast cereals have consistently been the top sources of WG intake, accounting for about 50 % of the WG in the diet. As a source of WG, RTE breakfast cereals have decreased for children age 6–18 between 2001 and 02 and 2011–12 (39.6 to 27.1 %) (Table 4). Conversely, yeast breads provided a much larger percentage of WG in children’s diet in 2011–12 (34 %) than in 2001–02 (17 %). Since 2001–02, crackers and salty grain snacks have increased as a source of WG for children. These foods provided 6.8 % of the WG intake in 2001–02 and 9.8 % of the intake in 2011–12. Popcorn and oatmeal as a source of WG have decreased over time, and each account for 5–10 % of the WG intake in children. In adults, yeast bread, crackers and salty grain snacks have increased as a source of WG over the 12 year time period (Table 4). Comparing 2001–02 and 2011–12 cycle years, notable decreases were seen in the WG contribution from RTE breakfast cereals (25.4 vs. 18.6 %), popcorn (11.1 vs. 5.3 %) and oatmeal (13.6 vs. 10.6 %).

**Table 4:** Percent WG intake from different food groups in children (6–18 years) and adults (19+ years) based on NHANES (2001–02 to 2011–12)^a,b^

Children (6–18 years)	2001–02 (*n* = 3147)	2003–04 (*n* = 2747)	2005–06 (*n* = 2838)	2007–08 (*n* = 2134)	2009–10 (*n* = 2263)	2011–12 (*n* = 2151)
Food	Percent (%)^b^					
Yeast breads, rolls	17.7	20.4	20.0	25.1	26.5	34.0
RTE cereals	39.6	34.4	33.1	29.4	26.1	27.1
Popcorn	13.4	19.3	12.9	11.0	11.6	10.4
Crackers and non-popcorn salty snacks from grains	6.8	8.2	15.3	11.1	13.5	9.8
Cakes, cookies, pies, pastries	5.5	6.2	5.0	5.0	4.8	4.8
Oatmeal	9.7	3.7	5.2	9.2	4.5	4.4
Pastas, macaroni, rice	1.2	1.0	2.2	2.9	4.3	3.8
Grain mixtures, frozen plate meals, soups, meat substitutes	0.2	0.2	1.0	1.7	3.2	2.0
Pancakes, waffles, french toast, crepes	4.0	4.9	1.4	1.4	2.2	2.0
Quick breads	0.4	0.6	2.8	2.6	2.9	1.1
All other foods	1.1	1.1	0.6	0.6	0.3	0.7
Other cooked cereals	0.5	0.0	0.4	0.1	0.1	0.0
Flour and dry mixes	0.0	0.0	0.0	0.0	0.0	0.0
Total WG	100.0	100.0	100.0	100.0	100.0	100.0
Adults (19+ years)	2001–02 (*n* = 4647)	2003–04 (*n* = 4456)	2005–06 (*n* = 4405)	2007–08 (*n* = 5476)	2009–10 (*n* = 5820)	2011–12 (*n* = 4878)
Food	Percent (%)^b^					
Yeast breads, rolls	29.3	25.1	26.0	28.3	29.3	38.6
RTE cereals	25.4	23.6	25.1	24.4	23.1	18.6
Crackers and non-popcorn salty snacks from grains	8.1	7.5	13.3	11.9	10.5	12.0
Oatmeal	13.6	16.8	11.5	12.6	11.3	10.6
Pastas, macaroni, rice	2.8	3.0	3.4	4.6	4.7	5.9
Popcorn	11.1	11.1	7.9	7.3	7.9	5.3
Cakes, cookies, pies, pastries	3.5	4.2	3.8	4.1	2.7	3.2
Grain mixtures, frozen plate meals, soups, meat substitutes	0.4	0.9	3.2	1.1	2.1	2.4
Pancakes, waffles, french toast, crepes	3.0	4.0	1.4	0.5	1.0	1.1
All other foods	0.9	1.1	0.7	0.4	0.4	1.1
Quick breads	1.3	2.2	3.4	4.6	6.4	1.0
Other cooked cereals	0.4	0.5	0.3	0.1	0.7	0.2
Flour and dry mixes	0.0	0.0	0.0	0.0	0.0	0.0
Total WG	100.0	100.0	100.0	100.0	100.0	100.0

^a^Day 1 from foods only, excluding pregnant and breastfeeding women

^b^Mean WG intake from different food sources were estimated using PROC SURVEYMEANS and percent WG contribution from different food groups were calculated

Whole grain consumers had significantly better intakes of nutrients and other dietary components compared to non-consumers (Tables 5 and 6). Children age 6–18 who consumed some WG (0 < WG < 1 oz eq) had higher intakes of dietary fiber, potassium, calcium, vitamin C, B-vitamins, iron, zinc, magnesium, folate and vitamins A and D. Higher levels of WG consumption (at least 1 oz eq) also corresponded with overall improved nutrient intakes, but more specifically higher intakes of carbohydrate, protein, total sugars, vitamin E and selenium compared to lower WG intake. In addition, both children and adults who consumed WG had lower intakes of cholesterol. In the adult population, those who consumed at least 1 oz eq/day had higher intakes of carbohydrate, protein, total sugars, dietary fiber, potassium, calcium, thiamin, riboflavin, vitamin B12, iron, magnesium, folate, selenium, and vitamins E, A, D and C. These increases in nutrient intake could be partially explained by increased total energy intake. Energy intake for both adults and children were higher for those consuming at least 1 oz eq compared to no WG (0 oz eq/day) and low (>0 to <1 oz eq/day) consumers.

**Table 5:** Mean nutrient intake across WG consumption groups in children (6–18 years) based on NHANES (2001–2012)^1,2^

	WG consumption groups			*P* value
Energy/nutrient	WG = 0 (*n* = 7768)	0 < WG < 1 (*n* = 4675)	WG > =1 (*n* = 2837)	
Energy (kcal)	2060 ± 16^a^	2045 ± 19^a^	2292 ± 28^b^	<0.0001
Carbohydrate (g)	272 ± 2^a^	277 ± 2^a^	314 ± 4^b^	<0.0001
Total fat (g)	77.6 ± 0.8^a^	74.8 ± 0.9^b^	82.3 ± 1.3^c^	<0.0001
Protein (g)	71.9 ± 0.7^a^	71.4 ± 0.8^a^	81 ± 1.2^b^	<0.0001
Total sugars (g)	137 ± 1^a^	140 ± 2^a^	150 ± 2^b^	<0.0001
Total saturated fatty acids (g)	26.9 ± 0.3^a^	26.4 ± 0.4^a^	28.4 ± 0.5^b^	0.0012
Total polyunsaturated fatty acids (g)	15.5 ± 0.2^a^	14.9 ± 0.2^b^	17.8 ± 0.3^c^	<0.0001
Total monounsaturated fatty acids (g)	28.7 ± 0.3^a^	27.3 ± 0.4^b^	29.2 ± 0.5^a^	0.0008
Dietary fiber (g)	11.9 ± 0.2^a^	13.3 ± 0.2^b^	18.4 ± 0.3^c^	<0.0001
Cholesterol (mg)	244 ± 4^a^	220 ± 4^b^	230 ± 5^b^	0.0014
Sodium (mg)	3231 ± 35^a^	3230 ± 45^a^	3518 ± 53^b^	<0.0001
Potassium (mg)	2148 ± 25^a^	2244 ± 27^b^	2607 ± 44^c^	<0.0001
Calcium (mg)	932 ± 12^a^	1051 ± 14^b^	1218 ± 21^c^	<0.0001
Vitamin C (mg)	74.9 ± 2^a^	80.8 ± 2^b^	92.6 ± 4^c^	<0.0001
Thiamin (Vitamin B1) (mg)	1.5 ± 0.02^a^	1.6 ± 0.02^b^	2 ± 0.03^c^	<0.0001
Riboflavin (Vitamin B2) (mg)	1.9 ± 0.03^a^	2.2 ± 0.03^b^	2.5 ± 0.04^c^	<0.0001
Niacin (mg)	20.8 ± 0.3^a^	22 ± 0.3^b^	26.5 ± 0.4^c^	<0.0001
Vitamin B6 (mg)	1.6 ± 0.03^a^	1.7 ± 0.03^b^	2.2 ± 0.05^c^	<0.0001
Vitamin B12 (mcg)	4.7 ± 0.1^a^	5.3 ± 0.1^b^	6.1 ± 0.2^c^	<0.0001
Iron (mg)	13.2 ± 0.1^a^	15.1 ± 0.2^b^	19.7 ± 0.3^c^	<0.0001
Zinc (mg)	10 ± 0.1^a^	11.3 ± 0.1^b^	13.7 ± 0.2^c^	<0.0001
Magnesium (mg)	216 ± 2^a^	233 ± 2^b^	306 ± 4^c^	<0.0001
Folate, DFE (mcg)	473 ± 7^a^	579 ± 9^b^	713 ± 17^c^	<0.0001
Vitamin E as alpha-tocopherol (mg)	6.1 ± 0.1^a^	6.2 ± 0.1^a^	7.7 ± 0.2^b^	<0.0001
Vitamin A, RAE (mcg)	504 ± 10^a^	621 ± 10^b^	740 ± 18^c^	<0.0001
Vitamin D (D2 + D3) (mcg)	4.8 ± 0.1^a^	5.9 ± 0.1^b^	6.4 ± 0.2^c^	<0.0001
Selenium (mcg)	96 ± 1.1^a^	95.6 ± 1.2^a^	111.1 ± 1.6^b^	<0.0001

^1^Day 1 from foods only, excluding pregnant and breastfeeding women

^2^Mean nutrient intake was estimated using PROC SURVEYMEANS and ANOVA was used to compare the mean nutrient intake between three WG consumption groups, means with different superscript letters across rows are significantly different.

**Table 6:** Mean nutrient intake across WG consumption groups in adults (19+ years) based on NHANES (2001–2012)^1, 2^

	WG consumption groups			*P* value
Energy/nutrient	WG = 0 (n = 14,742)	0 < WG < 1 (*n* = 7479)	WG > =1 (*n* = 7462)	
Energy (kcal)	2177 ± 15^a^	2079 ± 14^b^	2285 ± 17^c^	<0.0001
Carbohydrate (g)	259 ± 2^a^	251 ± 2^b^	289 ± 2^c^	<0.0001
Total fat (g)	82.6 ± 0.7^a^	80 ± 0.6^b^	83.7 ± 0.8^a^	0.0002
Protein (g)	82.1 ± 0.6^a^	79.5 ± 0.6^b^	88.3 ± 0.7^c^	<0.0001
Total sugars (g)	122 ± 1.2^a^	118 ± 1.4^b^	127 ± 1.5^c^	<0.0001
Total saturated fatty acids (g)	27.4 ± 0.3^a^	26.2 ± 0.2^b^	26.8 ± 0.3^ab^	0.0029
Total polyunsaturated fatty acids (g)	17.2 ± 0.2^a^	17.4 ± 0.2^a^	19.3 ± 0.2^b^	<0.0001
Total monounsaturated fatty acids (g)	30.6 ± 0.3^a^	29.3 ± 0.2^b^	30.2 ± 0.3^a^	0.0016
Dietary fiber (g)	13.7 ± 0.2^a^	15.5 ± 0.1^b^	22 ± 0.2^c^	<0.0001
Cholesterol (mg)	303 ± 3^a^	278 ± 3^b^	267 ± 4^c^	<0.0001
Sodium (mg)	3501 ± 25^a^	3369 ± 28^b^	3700 ± 30^c^	<0.0001
Potassium (mg)	2556 ± 18^a^	2686 ± 19^b^	3099 ± 23^c^	<0.0001
Calcium (mg)	858 ± 9^a^	923 ± 9^b^	1106 ± 11^c^	<0.0001
Vitamin C (mg)	79.3 ± 1.6^a^	88.7 ± 1.8^b^	101 ± 2.1^c^	<0.0001
Thiamin (Vitamin B1) (mg)	1.5 ± 0.01^a^	1.6 ± 0.02^b^	2 ± 0.02^c^	<0.0001
Riboflavin (Vitamin B2) (mg)	2 ± 0.02^a^	2.2 ± 0.02^b^	2.6 ± 0.02^c^	<0.0001
Niacin (mg)	23.7 ± 0.2^a^	24.2 ± 0.2^a^	28.6 ± 0.2^b^	<0.0001
Vitamin B6 (mg)	1.8 ± 0.02^a^	1.9 ± 0.02^b^	2.4 ± 0.02^c^	<0.0001
Vitamin B12 (mcg)	4.9 ± 0.1^a^	5.5 ± 0.2^b^	6.1 ± 0.1^c^	<0.0001
Iron (mg)	13.5 ± 0.1^a^	15.1 ± 0.1^b^	19.7 ± 0.2^c^	<0.0001
Zinc (mg)	11.2 ± 0.1^a^	11.6 ± 0.1^b^	14.1 ± 0.1^c^	<0.0001
Magnesium (mg)	263 ± 2^a^	284 ± 2^b^	368 ± 3^c^	<0.0001
Folate, DFE (mcg)	473 ± 4^a^	540 ± 6^b^	687 ± 8^c^	<0.0001
Vitamin E as alpha-tocopherol (mg)	7 ± 0.1^a^	7.6 ± 0.1^b^	9.1 ± 0.1^c^	<0.0001
Vitamin A, RAE (mcg)	522 ± 9^a^	657 ± 18^b^	787 ± 13^c^	<0.0001
Vitamin D (D2 + D3) (mcg)	3.9 ± 0.1^a^	4.9 ± 0.1^b^	5.8 ± 0.1^c^	<0.0001
Selenium (mcg)	108 ± 1^a^	107 ± 1^a^	121 ± 1^b^	<0.0001

^1^Day 1 from foods only, excluding pregnant and breastfeeding women

^2^Mean nutrient intake was estimated using PROC SURVEYMEANS and ANOVA was used to compare the mean nutrient intake between three WG consumption groups, means with different superscript letters across rows are significantly different.

Baseline age and weight characteristics for children and adults across NHANES cycles are shown in Table 7. Comparing sample means in children from 2001 to 02 to 2011–12, BMI increased from 20.9 to 21.3 kg/m^2^, WC increased from 73.1 to 74.1 cm, and the % OW/OB increased from 32.7 to 35.9 % with a larger increase in % OB from 16.5 to 20.5 %. Similar increasing trends in mean weight measures were seen for adults comparing across the 12 year timeframe, in particular an increase in the % OB from 30.5 to 34.6 %. In adults, there was also an increase in mean age from 45.6 to 46.9 years that could reflect the increasing longevity of the US population (Table 7). Results from multiple regression analysis (Table 8) showed a significant, inverse relationship between BMI and WC with respect to WG intake in both children and adults after adjustment for covariates (*p* < 0.05). Logistic regression analysis showed children consuming at least 1 oz eq WG were significantly less OW/OB and OB compared to those with no WG intake. A similar, significant inverse relationship between percent OW/OB (*p* < 0.0001) and OB (*p* = 0.0035) with increasing WG consumption was also observed for adults (Table 8).

**Table 7:** Baseline age and weight characteristics for children (6–18 years) and adults (19+ years) from NHANES (2001–02 to 2011–12)^a^

Age group	2001–2002	2003–2004	2005–2006	2007–2008	2009–2010	2011–12
6–18 years	Mean ± standard error (5th, 95th Percentile)^b^					
*n*	3147	2747	2838	2134	2263	2151
Age (years)	12.0 ± 0.1 (6,18)	12.1 ± 0.2 (6,18)	12.1 ± 0.2 (6,18)	12.1 ± 0.1 (6,18)	12.0 ± 0.1 (6,18)	11.9 ± 0.1 (6,18)
BMI (kg/m^2^)	20.9 ± 0.2 (15,31)	21.1 ± 0.3 (15,31)	21.1 ± 0.3 (15,32)	21.1 ± 0.2 (15,32)	21.3 ± 0.2 (15,32)	21.3 ± 0.2 (15,32)
Waist circumference (cm)	73.1 ± 0.4 (54,102)	73.6 ± 0.7 (53,101)	74.0 ± 0.9 (53,105)	73.5 ± 0.6 (53,104)	73.7 ± 0.4 (53,103)	74.1 ± 0.6 (53,103)
Overweight/obese (%)^c^	32.7	34.8	32.6	35.1	33.7	35.9
Obese (%)^c^	16.5	17.2	17.5	18.8	19.1	20.5
19+ years	Mean ± standard error (5th, 95th Percentile)^b^					
*n*	4647	4456	4405	5477	5820	4878
Age (years)	45.6 ± 0.5 (21,77)	46.5 ± 0.5 (21,78)	46.9 ± 0.7 (21,78)	46.4 ± 0.5 (21,78)	46.8 ± 0.5 (21,78)	46.9 ± 1.0 (21,79)
BMI (kg/m^2^)	28.0 ± 0.2 (20,40)	28.1 ± 0.1 (24,39)	28.5 ± 0.3 (20,41)	28.6 ± 0.2 (20,41)	28.7 ± 0.1 (20,41)	28.6 ± 0.2 (20,41)
Waist circumference (cm)	96.0 ± 0.4 (73,124)	97.2 ± 0.3 (75,125)	97.4 ± 0.7 (73,126)	97.8 ± 0.5 (74,127)	98.1 ± 0.4 (74,127)	98.4 ± 0.6 (76,128)
Overweight/obese (%)^c^	65.1	65.6	66.6	67.7	68.9	67.7
Obese (%)^c^	30.5	32.3	33.3	33.9	36.2	34.6

^a^Day 1 from foods only, excluding pregnant and breastfeeding women

^b^Means, 5th and 95th percentile were calculated using PROC SURVEYMEANS for all the 2 year cycles

^c^% Overweight/Obese and % Obese were calculated using PROC SURVEYFREQ for all the 2 year cycles

**Table 8:** Adiposity measures across WG consumption groups based on NHANES (2001–2012)^1-6^

Age group and measure	WG consumption groups			*P* value
	WG = 0 (*n* = 7768)	0 < WG <1 (*n* = 4675)	WG > =1 (*n* = 2837)	
6–18 years				
Weighted %	47	32	21	
BMI (kg/m^2^)^2^	21.5 ± 0.2^a^	21.2 ± 0.2^b^	20.9 ± 0.2^c^	0.0004
Waist circumference (cm)^2^	74.0 ± 0.3^a^	73.1 ± 0.3^b^	72.6 ± 0.4^b^	0.0057
Overweight/obese (%)^3^	35.4^a^	32.9^ab^	30.3^b^	0.0210
Obese (%)^4^	19.4^a^	17.6^ab^	15.6^b^	0.0232
	WG = 0 (*n* = 14,742)	0 < WG <1 (*n* = 7479)	WG > =1 (*n* = 7462)	
19+ years				
Weighted percent (%)	47	26	27	
BMI (kg/m^2^)^2^	28.3 ± 0.1^a^	28.1 ± 0.1^a^	27.6 ± 0.1^b^	<0.0001
Waist circumference (cm)^2^	96.6 ± 0.3^a^	95.7 ± 0.3^b^	94.7 ± 0.3^c^	<0.0001
Overweight/obese (%)^3^	66.2^a^	66.5^a^	62.3^b^	<0.0001
Obese (%)^4^	33.0^a^	31.0^a^	28.1^b^	0.0035

^1^Day 1 from foods only, excluding pregnant and breastfeeding women

^2^Multivariate linear regression was performed to determine the extent to which adiposity measures (BMI, and waist circumference) were explained by WG intake

^3^Multivariate logistic regression was performed to estimate how the likelihood of being overweight/obese differs across WG consumption groups

^4^Multivariate logistic regression was performed to estimate how the likelihood of being Obese differs across WG consumption groups

^5^Regression analysis was adjusted for age, age^2^, gender, race/ethnicity, total calorie intake, alcohol intake and physical activity^6^Means with different superscript letters across rows are significantly different.

## Discussion

The examination of trends in TG intake, WG intake, food sources, and effects on nutrient intake and adiposity measures provides evidence for small changes in intake over 12 years, shifts in dietary sources, and confirmation that WG foods contribute to dietary quality and weight management. Dietary guidelines have consistently highlighted the importance of grains as a staple food group, but have more recently emphasized increasing the proportion consumed as WG and decreasing the proportion consumed as refined grain [1]. Consumption of TG has slightly decreased in children since 2001–02, and although adult TG intake decreased from 2001 to 02 through 2007–08, it rebounded back to similar consumption levels in 2011–12. These findings indicate that little progress in decreasing intake of refined grains has occurred in the past 12 years.

Small increases in WG intake were observed over 12 years, with intake in the last 2 year cycle (2011–2012) higher than all previous cycles. However, the proportion of the population who met WG recommendations in 2011–12 was still <1 % for children (6–18 y) and <10 % for adults. Therefore, the ability to achieve the potential health benefits associated with intake of 40–50 g WG/d is limited [2]. Low levels of consumption have also been reported for individuals of varying ages in France [44], Germany [45], Ireland [46], and the United Kingdom [47]. Intake by French children and adults was estimated to be about 9.1 and 14.4 g/d, respectively [44] and intake by children/teenagers and adults in the United Kingdom was estimated to be 13 and 20 g/d, respectively [47]. A longitudinal study of German children and adolescents reported WG intakes of 20 and 33 g/d, respectively, from weighed dietary records [45], and a national study of Irish children and teens using 7 day food records showed an intake of 18.5 and 23.2 g/d, respectively [46]. These low intake levels indicate that in spite of the differences in whole grain sources and eating contexts across the U.S. and Europe, meeting recommendations for WG intake is a widespread challenge.

The continued low percentage of children (6–18 y) and adults (19+ y) in the U.S. not meeting WG recommendations is unexpected given the increased reported availability of WG foods in the marketplace [48] and in schools based on new school meal requirements [49]. Since the 2005 Dietary Guidelines release, WG pasta, bread and cereal introductions have increased and growth has been observed in WG product sales volume, and in whole or multi-grain bread sales relative to all bread sales [48]. Alignment of food assistance programs with the Dietary Guidelines for Americans [49] which occurred in 2009 for the WIC program and in 2012 for the School Lunch and Breakfast Programs may facilitate a future increase in availability of WG foods and therefore the proportion of Americans meeting WG recommendations. WIC served 8.3 million participants in 2014 [50] and the School Lunch and Breakfast Programs served 30.4 and 13.6 million participants, respectively in 2014 [51]. As the proportion of whole grain-rich foods served in schools increases, the proportion of TG intake consumed as refined grain by children may decrease.

Several studies support the important contributions that breakfast and RTE breakfast cereal make to WG intake. Data from NHANES 2001–10 and the national Continuing Survey of Food Intake by Individuals 1989–1991 showed that breakfast eating occasions accounted for a large proportion of WG intake (39–53 %) [29, 52]. Children (5–18 y) participating in the third School Nutrition Dietary Assessment Study (2004–2005) who ate breakfast which included RTE cereal had higher WG intake compared to those not eating breakfast [53]. In the current study, RTE breakfast cereals and oatmeal have decreased as a source of WG for children, while yeast breads have increased. Based on nationally representative samples of students in grades 6 to 10 surveyed during the 2001–2002 and 2009–2010 school years, eating breakfast on weekdays and weekends had significantly increased [54]. However, national intake data also indicated that energy intake from RTE cereals among children and adolescents decreased from 79 kcal/d (1989–91) to 56 kcal/d (2009–10) [55]. RTE cereals represented a lower proportion of both TG (6.9 to 5.0 %) and WG (39.6 to 27.1 %) intake for children in the current study from 2001 to 2012. Therefore, the types of foods served in schools or homes for breakfast may have changed over time in the interest of convenience, with children eating hand-held or portable foods instead of sitting down to eat a bowl of cereal.

For both adults and children from 2011 to 2012, grain mixtures including frozen meals accounted for more than 25 % of TG intake, but only about 2 % of WG intake. These findings indicate that grain mixtures may represent a category that could benefit from reformulation to include more whole grain and therefore impact WG intake in the future. Examples of grain products within this category include pasta, pizza, soups (eg, containing noodles or rice), egg rolls, and pizza rolls. In a previous modeling study, substitution of refined grain in a variety of product categories with a specified level of whole grain was expected to increase WG intake by 1.7 oz eq/day [56]. However, most of the projected increase was based on reformulated yeast bread/rolls and quick breads (0.85 oz eq/day) and less from foods representing grain mixtures (0.61 oz eq/day) including pizza and other mixtures containing flour, and rice and pasta (including those in mixtures). Reformulation of grain products to include whole grain is challenging for food manufacturers with respect to maintaining acceptable taste, texture and flavor, especially for product categories considered to be less traditional sources of WG [23]. The development of effective technological approaches to address these challenges may allow for a wider variety of whole grain foods to be developed in the future.

The results of the current study indicated that crackers and non-popcorn salty grain snacks have increased over the past 12 years as a source of WG for children while also accounting for a substantial proportion of TG intake. This finding is consistent with results from other studies showing an increased trend in snacking for U.S. children, and that savory/salty snacks are a popular, widely-available snacking choice for children/adolescents. An analysis of snacking trends among U.S. children showed that snacking frequency significantly increased from 1977 to 78 to 2003–06 by about 1 more snack/d and the proportion of total energy intake from snacks increased to 27% [57]. The analysis also showed that salty snacks (crackers, chips, popcorn, and pretzels) accounted for the second largest source of total energy from snacks in 2006 [57]. Savory snacks (potato and other starchy vegetable chips, popcorn, pretzels, rice crackers, savory crackers, and zwieback toast) were consistently one of the seven major food sources contributing to energy intake for U.S. children/adolescents from 1989 to 2010 [55]. Finally, snack foods are widely available for purchase by children/adolescents in schools, retail food and non-food retail environments and restaurants according to a recent review on child snacking patterns [58].

In the current study, children and adults who consumed the highest amount of WG (>1 oz eq/d) had higher intakes of shortfall nutrients (vitamin A, vitamin D, vitamin E, folate, vitamin C, calcium, and magnesium) [20] as well as energy, total sugars and sodium compared with those who consumed low (0 < WG < 1 oz eq/d) or no WG intake/d. These findings indicate that careful selection of WG foods by consumers is warranted to avoid those foods with higher sugar and sodium content. The findings with respect to greater energy, fiber and magnesium intakes with greater intake of WG are consistent with previous studies for both children (6–18 y) and adults (19+ y) [17, 18]. Contrary to the findings in the current study, O’Neil et al. [17, 18] observed either lower or no significant differences in intakes of sodium and total sugars with greater intakes of WG. However, the categories of WG intake were based on different cutpoints compared to the current study which may account for the lack of consistency between studies.

Recent reviews have indicated inverse associations between WG intake and BMI, waist circumference, and weight gain among adults in epidemiological studies [9, 59] consistent with findings from the current study. Findings from the current study were also consistent with a previous cross-sectional study involving NHANES 1999–2004 for adolescents based on an inverse relationship between WG intake and BMI z-score and BMI-for-age percentile [16]. However, WG intake as part of an energy-restricted diet has not been effective in reducing body weight in short-term, randomized-controlled, intervention studies among adults or children [7, 60]. Giacco et al. [59] suggests that intervention study results may have been affected by methodological issues including small numbers of study participants and short duration explaining in part the lack of consistency of results compared to epidemiology studies. Therefore, the results of the current study may not be directly applicable in clinical settings. Further intervention studies addressing these limitations are needed among both adults and children.

A number of dietary and physical factors may explain the inverse associations observed between WG intake and lower body weight in epidemiological studies. Giacco et al. [59] suggest possible dietary mechanisms including lower energy density of WG products and lower glycemic index of WG compared to refined grain foods which may enhance satiety. However, in the current study among adults and children, both energy and fiber intakes were higher while BMI, waist circumference, % OV/OB and % OB were lower in the highest WG consumption group compared to non-WG consumers. These findings do not support the dietary mechanisms suggested [59], therefore mechanisms other than effects on satiety may need to be examined.

The low intake of WG and small percentage of children and adults in the U.S. meeting WG recommendations indicates a need for attention to factors that affect selection and intake of WG foods by consumers [23]. Information about WG content could be made available on WG food packages in response to reports that this information is difficult to interpret [61]. For example, the ability to identify WG foods was identified as a problem in purchasing WG foods for schools by food service managers [61]. A standard definition of WG foods has also been suggested as a means to enable health professionals and educators to better promote increased intake [62].

Strengths of the current study include the use of 6 2-year cycles (2001–02, 2003–04, 2004–06, 2007–08, 2009–10, 2011–12) of NHANES data which increases the number and types of foods included in the analysis. In addition, these data were obtained from a large, nationally representative sample. In terms of limitations, due to the cross-sectional nature of the NHANES study design, causal inferences cannot be made. The use of one day of dietary recalls is also a potential limitation as it may not reflect usual consumption; however, data from days 1 and 2 were used to determine the percentage of individuals meeting TG and WG recommendations. Ounce equivalent units for grain intake were used in the current study instead of reporting intake in g dry weight/day as recently suggested by Ross et al. [63]. However, because total and whole grain intake recommendations based on the U.S. Dietary Guidelines for Americans are made in oz eq [1], TG and WG intake were expressed in the same measure to enable evaluation of intake with respect to recommendations. Also, in the USDA food patterns, which are based on the Dietary Guidelines for Americans, one oz eq of grains can be either 16 grams or 28.35 g of grain, depending upon the food source [39]. Therefore in this case, back converting oz eq to grams could not be done in a meaningful way. Consistency in approaches to reporting WG consumption would allow for more meaningful comparisons in WG intake between datasets and across global regions. In addition, the current study reports a detailed description of total WG intake across food categories, but not by specific grain type. Based on the top food sources of WG intake, one could infer that wheat (yeast breads, RTE cereals), oats (RTE cereals, oatmeal), and corn (popcorn, RTE cereals) are the grain types most commonly consumed, but a more definitive assessment is needed. A recent study from the HELGA cohort in Scandinavia did include WG intake by grain type, highlighting rye and wheat as primary sources across Denmark, Sweden and Norway [10]. This study utilized a FFQ which typically contains much fewer unique food items to review and identify the primary grain type compared to the variety of foods typically reported in a 24 h recall.

## Conclusions

Over the past 12 years, WG consumption has modestly increased in the US population, while TG intake has slightly decreased in children but remained steady in adults. Despite an increase in consumer interest and availability of WG foods in the marketplace, less than 10 % of Americans meet daily dietary goals to consume at least half of all grains as WG, while most continue to meet daily recommendations for TG. Higher consumption of WG can be seen as an indicator of a healthier diet and lifestyle pattern, as WG intake was associated with improved nutrient intake and weight measures in children and adults. Nutrition policy, health professional and food industry efforts should continue to highlight the significant gap in WG consumption, and provide actionable, convenient and affordable options to allow consumers to more easily integrate WG choices into daily eating habits.

## Abbreviations

AMPM: automated multiple-pass method
BMI: body mass index
D: day
FNDDS: Food and Nutrient Database for Dietary Studies
FPED: Food Patterns Equivalents Database
MPED: My Pyramid Equivalents Database
NCHS: National Center for Health Statistics
NCI: National Cancer Institute
NHANES: National Health and Nutrition Examination Survey
OW/OB: overweight/obese
oz eq: ounce equivalents
RTE: ready to eat
TG: total grain
U.S.: United States
USDA: United States Department of Agriculture
WC: waist circumference
WG: whole grain
WIC: women, infants and children special supplemental program
Y: year
